# Ameliorative effects of Berberine chloride against 5-fluorouracil-induced cardiotoxicity in Sprague Dawley rats

**DOI:** 10.1038/s41598-025-12389-6

**Published:** 2025-08-02

**Authors:** Mirna Akram Labib, Omar S. Saeed, Samar H. ElSharkawy, Marwa S. Khattab, Hesham Y. El-Zorba, Khaled Abo-EL-Sooud

**Affiliations:** 1 Department of Pharmacology, Faculty of Veterinary Medicine, Cairo University, Giza, 12211 Egypt; 2 Department of Virology, Faculty of Veterinary Medicine, Cairo University, Giza, 12211 Egypt; 3 Department of Surgery, Anaesthesiology, and Radiology, Faculty of Veterinary Medicine, Cairo University, Giza, 12211 Egypt; 4 Department of Pathology, Faculty of Veterinary Medicine, Cairo University, Giza, 12211 Egypt

**Keywords:** 5-Fluorouracil, Berberine, Cardiotoxicity, Antioxidant, ECG, Gene expression, Drug discovery, Cardiology, Medical research, Oncology

## Abstract

5-Fluorouracil (5-FU) is the most commonly used chemotherapeutic agent for solid malignancies. Although it has crucial therapeutic effects, it ranks as the second most cardiotoxic antineoplastic agent. Berberine (BBR) is a quaternary benzylisoquinoline alkaloid with promising antioxidant properties. The current study aimed to assess the palliative effect of BBR on 5-FU-induced cardiotoxicity in rats. Fifty male Sprague Dawley rats were randomly divided into five groups: negative control, which received 2% DMSO orally (PO) for 2 weeks; cardiotoxic, which received a single intraperitoneal (IP) injection of 5-FU (150 mg/kg); groups 3 and 4, which received a single IP injection of 5-FU (150 mg/kg) followed by BBR (50 mg/kg and 100 mg/kg, respectively) PO for 2 weeks; and a BBR-only group, which received BBR (100 mg/kg) PO for 2 weeks. On the 14th day, all groups underwent ECG evaluation. Blood and heart samples were collected 24 h after the last dose for further investigations. 5-FU induced significant alterations in the ECG pattern and caused a significant increase in cardiac troponin I (cTnI), creatine kinase-MB (CK-MB), lactate dehydrogenase (LDH), and malondialdehyde (MDA). Moreover, it led to decreased levels of superoxide dismutase (SOD), reduced glutathione (GSH), and total antioxidant capacity (TAC). Our data suggest that BBR could mitigate 5-FU-induced cardiotoxicity by modulating cardiac injury markers, normalizing cTnI, CK-MB, and LDH levels, reducing oxidative stress by lowering MDA levels, and increasing SOD, GSH, and TAC levels. Additionally, BBR inhibited apoptotic events by suppressing caspase-3 activation and upregulating Bcl-2 expression, reduced the inflammatory response by downregulating cyclooxygenase-2 (COX-2) and tumor necrosis factor-alpha (TNF-α) expression levels, and decreased the risk of thrombosis by increasing endothelial nitric oxide synthase (eNOS) expression levels. In conclusion, BBR exerts ameliorative effects against 5-FU-induced cardiotoxicity in rats.

**Supplementary Information:**

The online version contains supplementary material available at 10.1038/s41598-025-12389-6.

## Introduction

Cancer is a major global public health threat and is the second leading cause of death in the United States^1^. In humans, the most frequent malignancies include those of the prostate, gastrointestinal tract, lungs, breast^2^, and blood^3^. A fatal adverse effect associated with antineoplastic drugs is cardiovascular damage to the heart muscle and/or valves, which develops gradually after treatment^4,5^. 5-Fluorouracil (5-FU) is among the most widely used anticancer agents approved by the US Food and Drug Administration (FDA) for the treatment of various solid tumors^6^. It has served as the cornerstone of systemic combination chemotherapy for head and neck cancer, breast cancer, colorectal cancer (CRC), and other gastrointestinal malignancies^7^. The incidence of 5-FU-induced cardiotoxicity ranges from 0 to 35%, depending on the chemotherapy regimen, dosage, and the presence of cardiac comorbidities^8^. Proposed mechanisms for this cardiotoxicity include endothelial damage followed by coagulation, oxidative stress-induced direct toxicity, activation of the inflammatory cascade, mitochondrial membrane damage, and increased thrombogenicity^9^. Clinically, it is associated with angina, pulmonary edema, congestive heart failure, myocardial infarction or ischemia, arrhythmias, and sudden cardiac arrest. Natural antioxidant compounds are commonly used to prevent or mitigate the toxic effects of 5-FU^10^.

Berberine (BBR), a non-basic, quaternary benzylisoquinoline plant alkaloid, has a long history of use in traditional Chinese and Ayurvedic medicine. The genus *Berberis*, belonging to the family *Berberidaceae*, comprises 450–500 species and represents the primary source of BBR^11^. It appears as a tasteless, deep yellow powder^12^. The BBR molecule consists of two benzene rings and one tetrahydropyran ring; one of the benzene rings bears a hydroxyl group (–OH), which is essential to its specific properties and biological activity^13^, and it is commonly available in the form of hydrochloride and sulfate salts^14^. Berberine exhibits potent antimicrobial and antioxidant activities. Numerous studies have reported its pharmaceutical benefits for the digestive, immune, and nervous systems, as well as for cardiovascular diseases^15^. Other pharmacological effects of BBR include anti-inflammatory^16^, hypotensive^17^, anticancer^18^, and antidiabetic activities^19^.

The purpose of this study was to investigate whether BBR treatment could reduce 5-fluorouracil-induced cardiotoxicity in rats and to elucidate its possible cardioprotective mechanisms.

## Results

### Effect of Berberine on ECG parameters

The ECGs of all groups were statistically analyzed and are summarized in Table 1. No significant differences were observed between the baseline ECG data and the group receiving BBR only. All other groups showed significant deviations from baseline measurements. Heart rate (HR) was significantly reduced in the 5-FU, 5-FU + BBR (50 mg/kg), and 5-FU + BBR (100 mg/kg) groups compared to the control group, with the highest HR recorded in the 5-FU + BBR (100 mg/kg) group. P wave amplitude was significantly higher in the 5-FU and 5-FU + BBR (50 mg/kg) groups than in the other groups, while no significant difference was observed among the remaining groups. A significant prolongation of the QRS complex and PR interval durations was observed in the 5-FU, 5-FU + BBR (50 mg/kg), and 5-FU + BBR (100 mg/kg) groups, with the shortest durations recorded in the 5-FU + BBR (100 mg/kg) group. P wave duration was significantly prolonged in the 5-FU and 5-FU + BBR (50 mg/kg) groups compared to the control group, while the other two groups showed no significant difference.

**Fig. 1:**
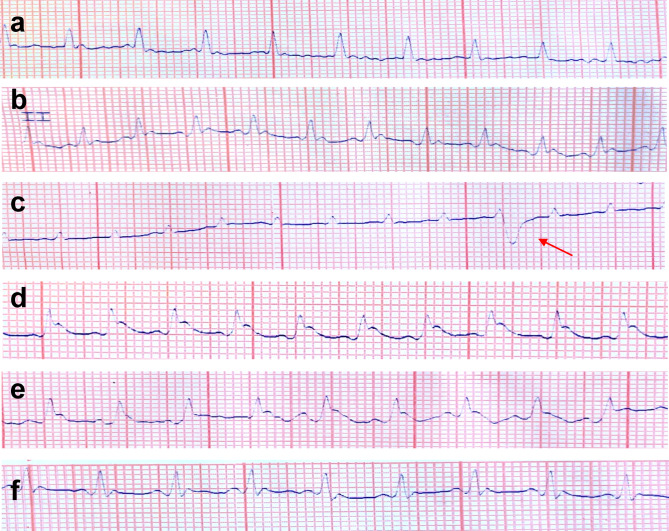
**a**–**f** Effect of 5-FU and BBR administration on ECG parameters in different groups. ECG Lead II readings of the study groups showing normal P-QRS-T morphology and rhythm in both control -ve (**a**) and BBR only groups (**b**). Both (**c**) and (**d**) represent the 5-FU group where wide QRS complex, elevated ST segment, and VPCs (red arrow) were detected, indicating the presence of cardiotoxicity. (**e**) The 5-FU + BBR (50 mg/kg) reading still showed an abnormal QRS complex and elevated ST segment. (**f)** Group 5-FU + BBR (100 mg/kg) showed significant improvement in the ECG readings by exhibiting normal QRS morphology and isoelectric ST segment, indicating the cardioprotective effect of BBR. All groups’ paper voltage settings were adjusted at 10 mm/mV except for **c** and **d**, which were fitted to 20 mm/mV and a 50 mm/s paper speed.

Heart rhythm was sinus and regular in all groups except for the 5-FU group, which exhibited sinus arrhythmia with transient left-sided ventricular premature beats. The ST segment was isoelectric in all groups except the 5-FU and 5-FU + BBR (50 mg/kg) groups, which showed either elevation or depression (Table 1; Fig. 1a–f).

**Table 1:** Effect of 5-FU and BBR administration on ECG parameters in different groups.

Groups	Control -ve	5-FU (150 mg/kg)	5-FU + BBR (50 mg/kg)	5-FU + BBR (100 mg/kg)	BBR (100 mg/kg)
Heart rate (bpm)	392.67 ± 25.60^a^	367 ± 23.28^b^	371 ± 24.25^b^	380 ± 30.80^c^	388 ± 25.60^a^
P amplitude (mV)	0.12 ± 0.01^a^	0.25 ± 0.02^b^	0.17 ± 0.01^c^	0.14 ± 0.01^a^	0.13 ± 0.01^a^
P duration (ms)	23.33 ± 5.77^a^	33.33 ± 5.77^b^	36.67 ± 5.77^c^	23.33 ± 5.77^a^	30.00 ± 00^a^
PR interval (ms)	39.25 ± 3.20^a^	62.41 ± 3.68^b^	47.33 ± 3.78^c^	42.08 ± 2.45^d^	40.67 ± 3.78^a^
QRS amplitude (mV)	0.47 ± 0.01^a^	0.21 ± 0.01^b^	0.56 ± 0.02^cd^	0.51 ± 0.01^c^	0.49 ± 0.01^a^
QRS duration (ms)	30 ± 00^a^	46.67 ± 5.77^b^	43.33 ± 5.77^c^	40 ± 00^d^	30 ± 00^a^
Heart rhythm	Sinus	Arrhythmia	Sinus	Sinus	Sinus
ST segment	Isoelectric	Variable		Isoelectric	Isoelectric
Abnormal observations	–	Multiple VPCs (left-sided)	—	—	—

All values are expressed as mean ± standard deviation (SD), *n* = 10. Using one-way ANOVA followed by Tukey post hoc test.

^a^, ^b^, ^c^, and ^d^indicate statistically significant differences compared to the control group (*p* < 0.05, *p* < 0.01, *p* < 0.001, and *p* < 0.0001, respectively). In the same row, the different letters mean significant differences between groups. While the same letters mean non-significant differences. VPCs = ventricular premature contractions.

### Effect of BBR on relative heart weight

**Fig. 2:**
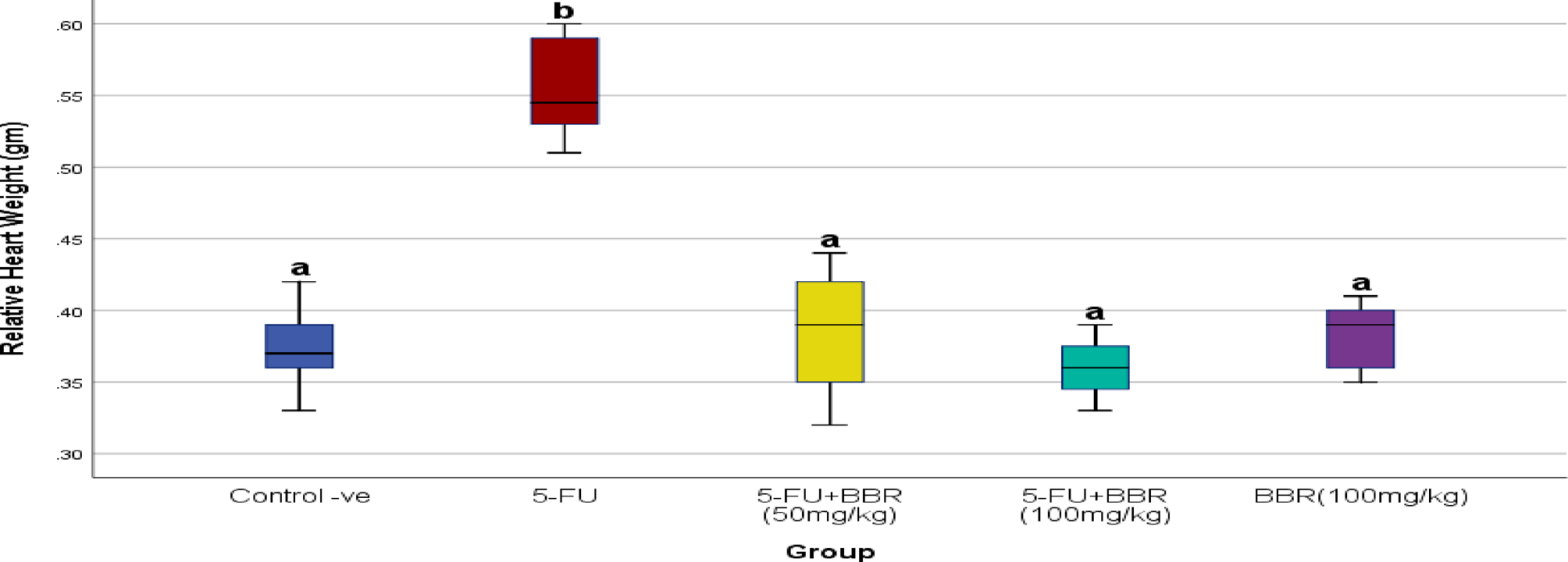
Effect of 5-FU and BBR administration on the relative heart weight of rats. All values are expressed as mean ± standard deviation (SD), *n* = 10. Using one-way ANOVA followed by Tukey post hoc test. ^a^ and ^b^ indicate (*p* < 0.05, *p* < 0.01, respectively).

The relative heart weight was significantly higher in the 5-FU group compared to all treatment groups (*p* < 0.01) (Fig. 2).

### Effect of BBR on hematological and biochemical parameters

#### Hematological parameters

**Fig. 3:**
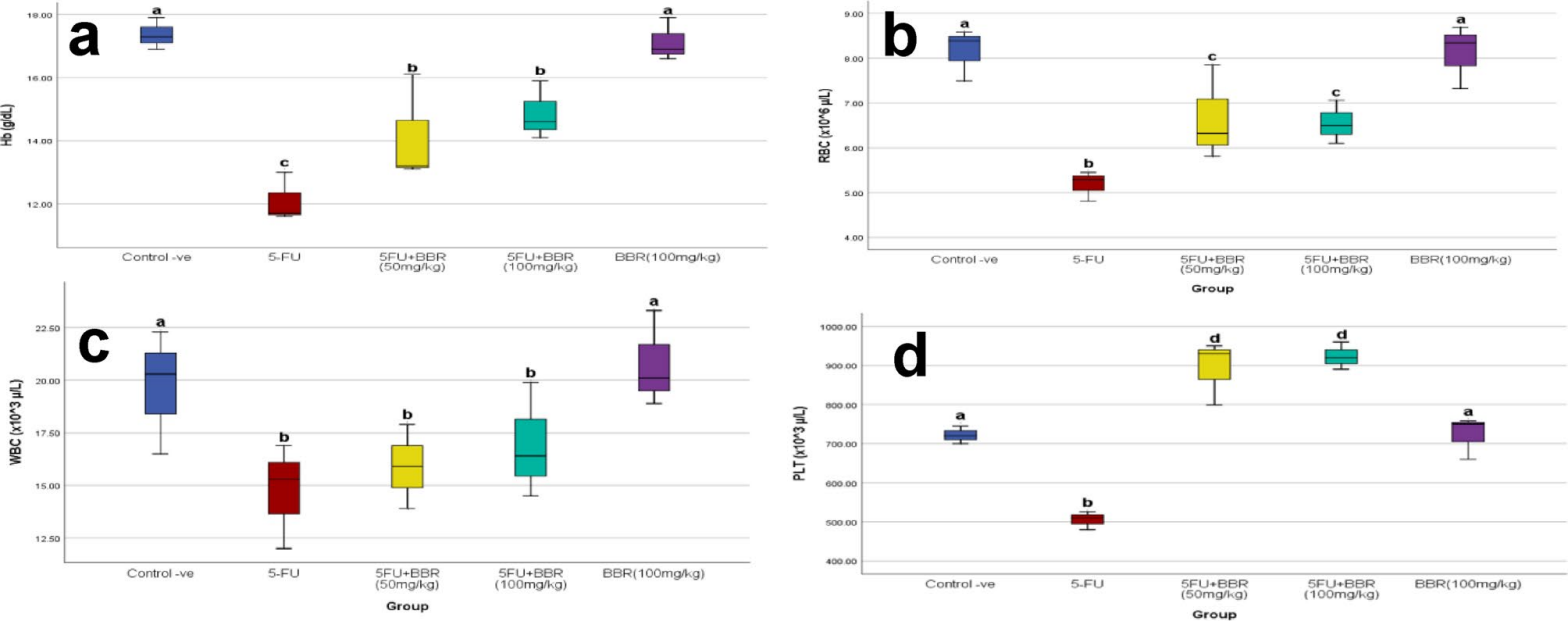
**a**–**d** Effect of 5-FU and BBR administration on hematological parameters. Hemoglobin (Hb, g/dL), red blood cells (RBC, × 10^6 µ/L) count, white blood cells count (WBC, × 10^3 µ/L), and platelet count (PLT, × 10^3 µ/L), *n* = 10. Using one-way ANOVA followed by Tukey post hoc test. ^a^, ^b^, ^c^, and ^d^ indicate statistically significant differences compared to the control group (*p* < 0.05, *p* < 0.01, *p* < 0.001, and *p* < 0.0001, respectively).

Rats that received 5-FU only showed significant decreases in hemoglobin (Hb) as well as red blood cell (RBC), white blood cell (WBC), and platelet counts. Meanwhile, BBR ameliorated these parameters, with similar effects observed at both doses (Fig. 3A–D).

#### Biochemical parameters

##### Cardiac toxicity markers

The activities of serum cardiac markers cTnI, CK-MB, and LDH were significantly elevated in 5-FU-treated rats by 66%, 96%, and 53%, respectively, indicating cardiac damage. The coadministration of BBR with 5-FU significantly reduced these cardiac markers compared to the 5-FU group. BBR alone did not alter these parameters compared to the control group, except for LDH levels, which were significantly reduced in the BBR-only group relative to the control negative group (Table 2).

##### Oxidative stress and antioxidant markers

5-FU induced a significant increase in lipid peroxide levels by 55% and significant decreases in cardiac antioxidant enzyme activities, TAC, GSH, and SOD, by 53%, 40%, and 20%, respectively. Meanwhile, the coadministration of 5-FU with BBR provided significant protection against 5-FU-induced oxidative stress by restoring MDA levels and the activities of antioxidant markers to normal levels (Table 2).

##### Liver function enzymes

Regarding liver function, rats treated with 5-FU showed significant alterations in hepatic transaminases ALT and AST by 14% and 48%, respectively, indicating moderate liver injury. Concomitant administration of BBR significantly restored liver function, with values approaching normal levels (Table 2).

**Table 2:** Effect of 5-FU and BBR administration on biochemical parameters.

Groups	Control -Ve	5-FU (150 mg/kg)	5-FU + BBR (50 mg/kg)	5-FU + BBR (100 mg/kg)	BBR (100 mg/kg)
cTn-1 (pg/ml, serum)	0.6 ± 0.10^a^	1.77 ± 0.25^c^	1.10 ± 0.17^b^	0.83 ± 0.15^a^	0.73 ± 0.15^a^
CK-MB (ng/ml, serum)	2.48 ± 0.65^a^	65.93 ± 15.06^d^	4.88 ± 1.30^a^	3.41 ± 0.71^a^	2.48 ± 0.82^a^
LDH (U/L, serum)	392.67 ± 37.58^a^	827.67 ± 54.26^d^	440.67 ± 23.86^b^	415.33 ± 34.79^a^	199 ± 27.62^c^
MDA (nmol/mg, tissue)	38 ± 2.65^a^	85.33 ± 10.07^d^	38.33 ± 10.50^a^	24.67 ± 5.77^c^	36 ± 4.58^a^
TAC (mmol/L, serum)	0.70 ± 1.00^a^	0.33 ± 0.06^c^	0.77 ± 0.15^a^	1.47 ± 0.06^b^	0.73 ± 0.06^a^
GSH (mM, serum)	6.07 ± 0.65^a^	3.60 ± 0.66^c^	7.30 ± 0.26^b^	11.47 ± 0.85^d^	7.33 ± 0.78^b^
SOD (U/gm, tissue)	87.37 ± 0.98^a^	70.20 ± 2.01^c^	85.73 ± 4.99^a^	96.13 ± 3.44^b^	85.77 ± 3.75^a^
ALT (U/L, serum)	37 ± 1.00^a^	43 ± 1.00^b^	34.73 ± 0.64^a^	35 ± 1.00^a^	37 ± 1.00^a^
AST (U/L, serum)	140 ± 10.54^a^	268.33 ± 28.45^d^	220.33 ± 9.50^c^	191.67 ± 7.64^b^	199.33 ± 9.02^b^

All values are expressed as Mean ± SD, *n* = 10. Using one-way ANOVA followed by Tukey post hoc test.

^a^, ^b^, ^c^, and ^d^ indicate statistically significant differences compared to the control group (*p* < 0.05, *p* < 0.01, *p* < 0.001, and *p* < 0.0001, respectively).

### Effect of BBR on histopathological and immunohistochemical changes in heart tissue

#### Histopathological findings

**Fig. 4:**
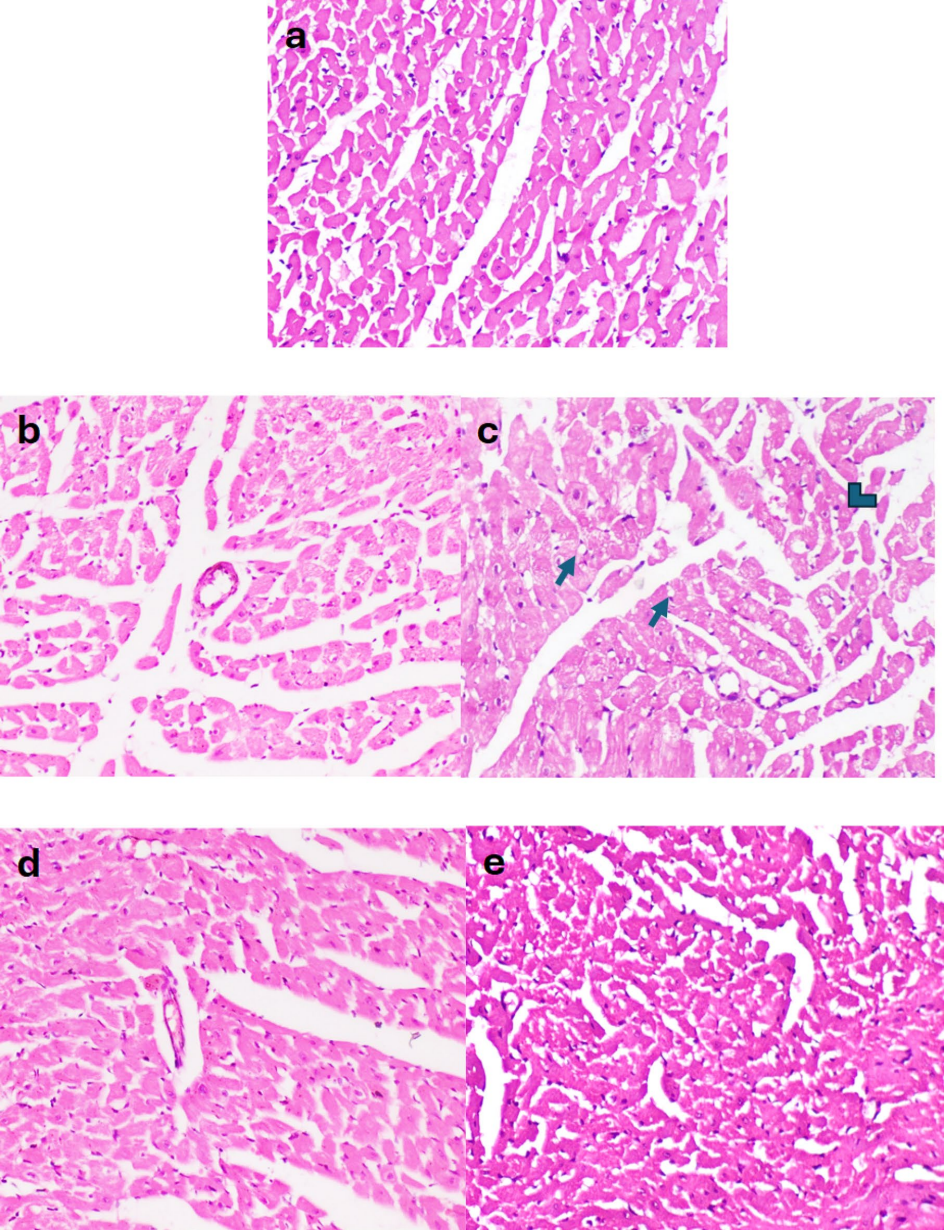
**a**–**e** Histopathology of the heart in different groups. **a** Normal histological structure in control negative and **b** BBR only, **c** vacuolation and necrosis in the cardiomyocytes (arrow), and mononuclear cells infiltration (arrowhead) between muscle bundles in 5-FU, **d** mild histopathological alteration in 5-FU + BBR (50 mg/kg) and **e** 5-FU + BBR (100 mg/kg). Hematoxylin and Eosin stain × 200.

In the control negative (Fig. 4a) and BBR-only groups (Fig. 4b), no histopathological alterations were observed in cardiomyocytes. In contrast, the 5-FU group exhibited vacuolation and necrosis of cardiomyocytes, along with infiltration of mononuclear and fibroblast cells between muscle bundles (Fig. 4c). In the treatment groups, 5-FU + BBR (50 mg/kg) and 5-FU + BBR (100 mg/kg), the histopathological lesions were notably reduced in a dose-dependent manner (Fig. 4d, e).

#### Immunohistochemical findings

**Fig. 5:**
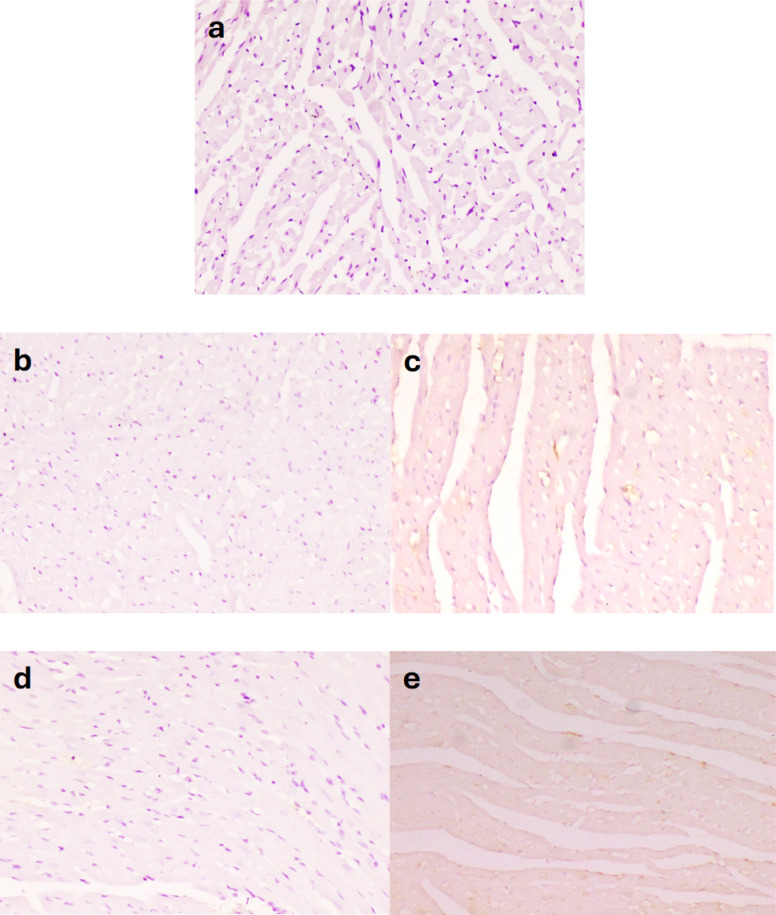
Microscopy of the heart in different groups. Weak caspase 3 expression was observed in **a** control negative and **b** BBR only, severe expression of caspase-3 in **c** 5-FU, weak expression in **d** 5-FU + BBR (50 mg/kg), and **e** 5-FU + BBR (100 mg/kg). Caspase-3 Immunoperoxidase × 200.

**Fig. 6:**
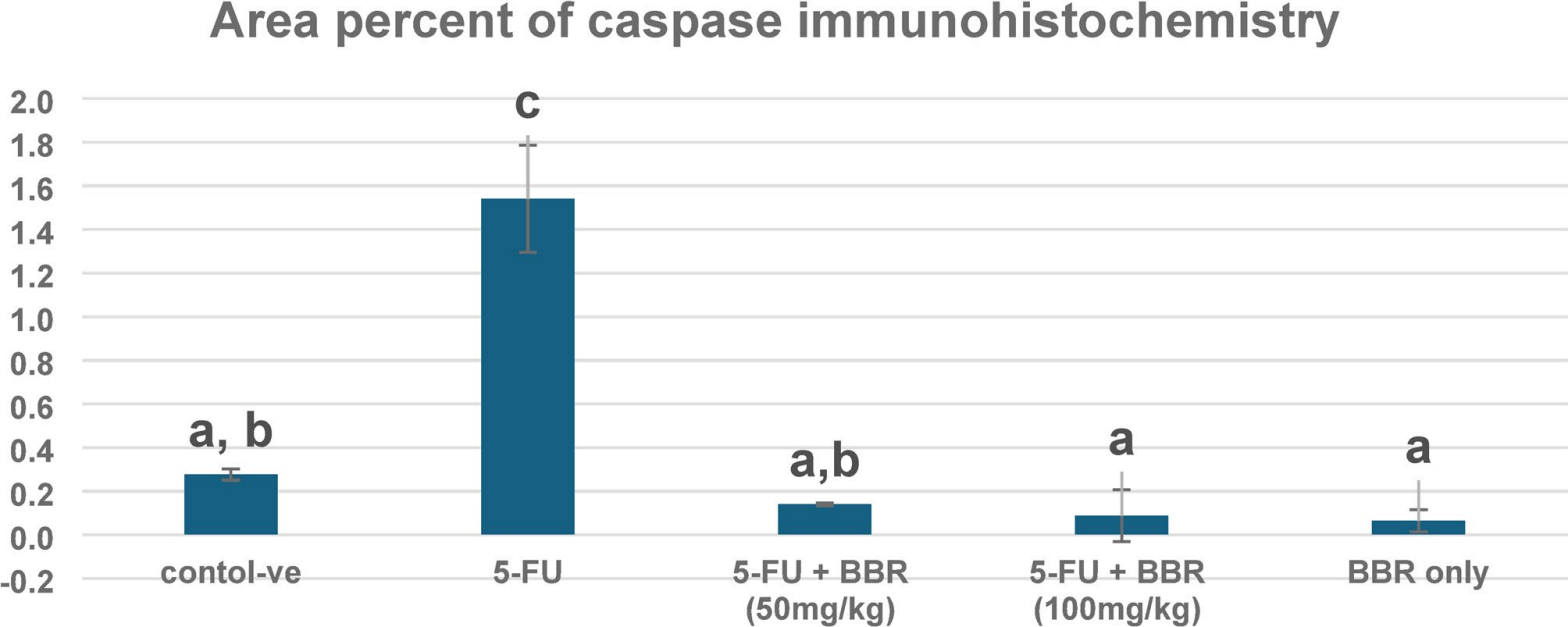
Area percent of caspase-3 immunohistochemistry in hearts of different groups. The data is expressed as mean value ± standard error, *n* = 10. Columns bearing different lowercase letters indicate significance at ^a^, ^b^, ^c^, and ^d^ indicate *p* < 0.05, *p* < 0.01, *p* < 0.001, and *p* < 0.0001, respectively.

Caspase-3 expression is considered an indicative marker of apoptotic cells. Caspase-3 expression in the control -ve group (Fig. 5a) and BBR-only group (Fig. 5b) was weak. The 5-FU-treated group exhibited a significant increase in caspase-3 expression in cardiac muscle, as indicated by intense brown staining (Fig. 5c). BBR-treated groups had a decrease in caspase-3 expression (Fig. 5d, e). Both doses of BBR significantly decreased the expression of caspase-3 in a dose-dependent manner (Fig. 6).

**Fig. 7:**
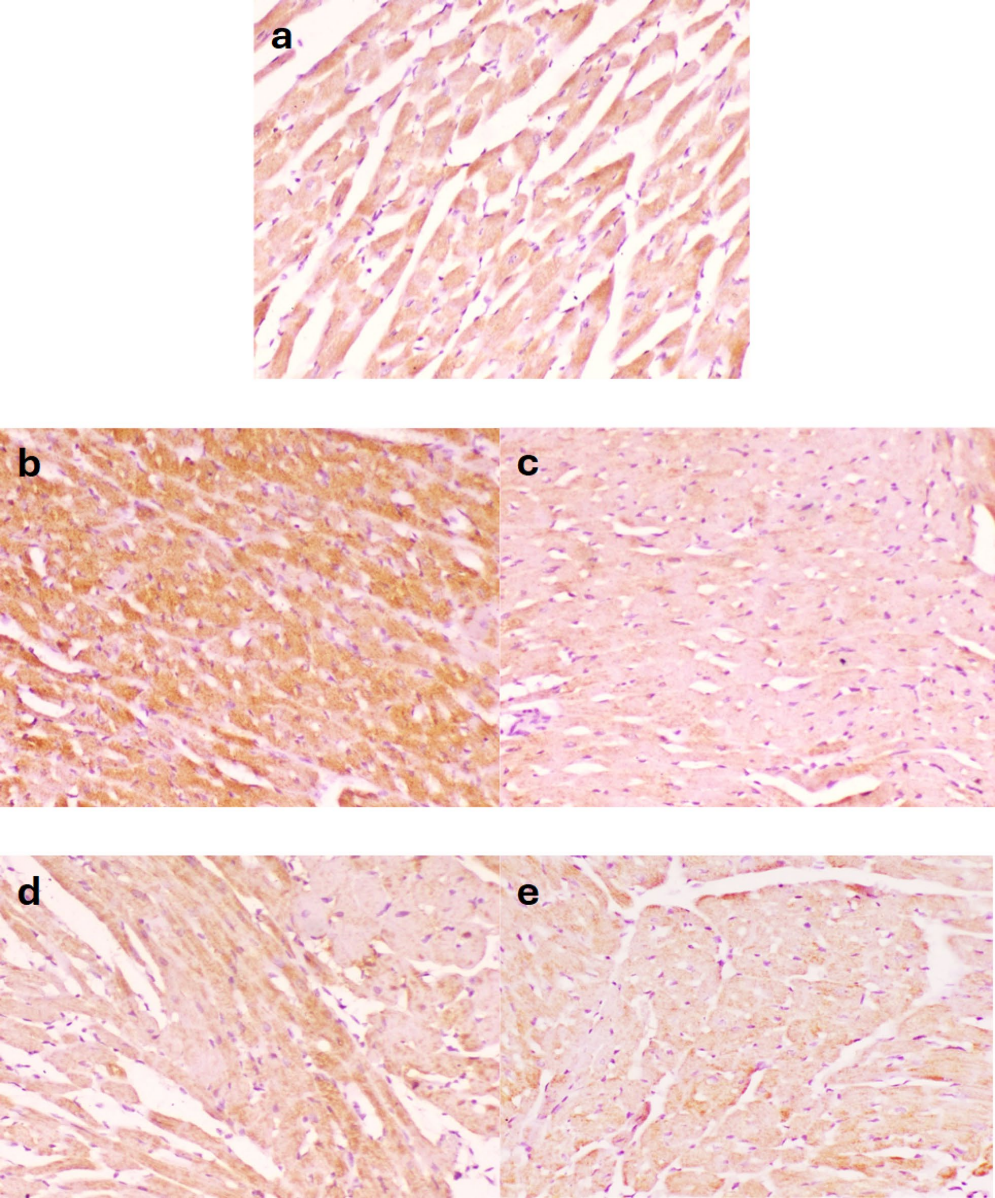
Microscopy of the heart in different groups. Severe expression of antiapoptotic Bcl2 protein in **a** control negative and **b** BBR only, weak expression of Bcl2 in **c** 5-FU, moderate expression of Bcl2 in **d** 5-FU + BBR (50 mg/kg) and **e** 5-FU + BBR (100 mg/kg). Bcl2 Immunoperoxidase × 200.

**Fig. 8:**
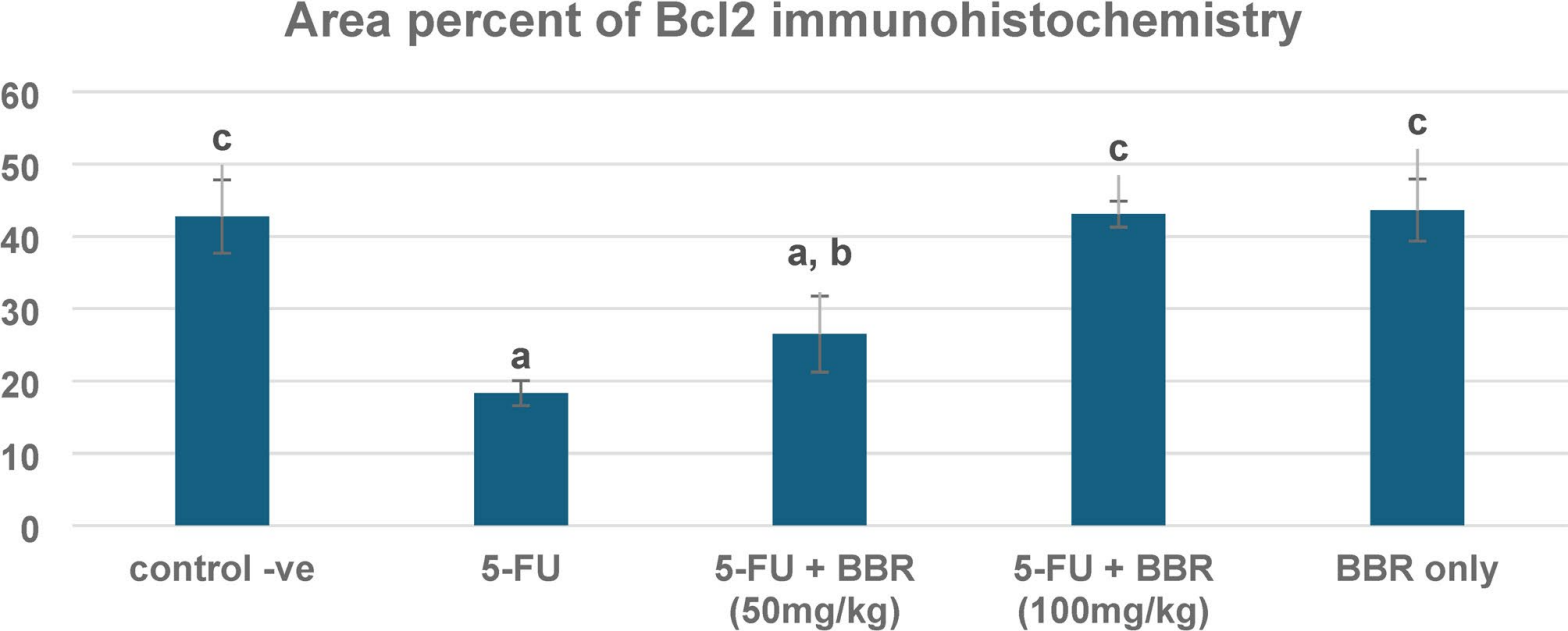
Area percent of Bcl-2 immunohistochemistry in hearts of different groups. The data is expressed as mean value ± standard error, *n* = 10. Columns bearing different lowercase letters indicate significance at ^a^, ^b^, ^c^, and ^d^ indicate *p* < 0.05, *p* < 0.01, *p* < 0.001, and *p* < 0.0001, respectively.

Bcl-2 was moderately expressed in the control -ve group (Fig. 7a) and BBR-only (Fig. 7b). The expression of antiapoptotic protein Bcl-2 was significantly decreased in 5-FU-treated rats (Fig. 7c). Conversely, concomitant administration of BBR significantly increased Bcl-2 expression in a dose-dependent manner (Fig. 7d, e), thereby diminishing the apoptotic machinery. The mean area percentage of Bcl-2 expression in different groups is represented in a chart (Fig. 8).

### Effect of BBR on *TNF-α*, *COX-2*, and *eNOS* gene expression

No amplification signals were detected in the no-RT control reactions during qPCR analysis, confirming the effectiveness of DNase I treatment and the specificity of the reverse transcription procedure.

#### *TNF‑α* expression

**Fig. 9:**
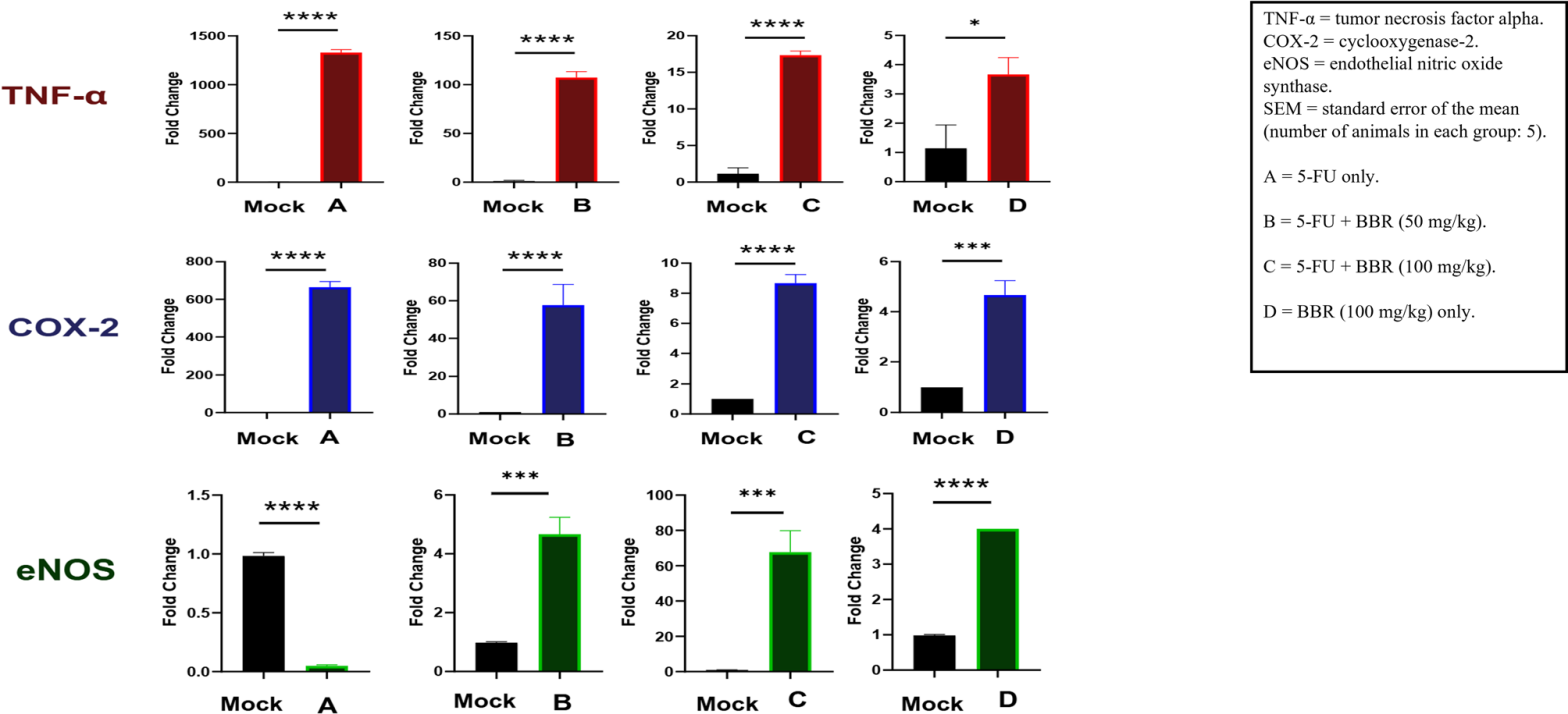
The effect of BBR administration on *TNF-α*, *COX-2*, and *eNOS* gene expression levels by modulating pro-inflammatory and anti-inflammatory cytokines in rats with cardiotoxicity. Gene expression levels of **A** *TNF-α*, **B** *COX-2*, and **C** *eNOS* were measured in heart tissues using quantitative real-time PCR and are presented as fold change relative to the control group. Data are shown as mean ± standard error of the mean (SEM), with *n* = 5 animals per group. Statistical significance was determined as *p* < 0.05 (*), *p* < 0.01 (**), *p* < 0.001 (***), and *p* < 0.0001 (****). The terms “mock” and “control–ve” both refer to the same group: untreated and unchallenged animals with 5-FU that served as the baseline reference group in the ΔΔCt analysis.

*TNF-α* expression was significantly elevated in the 5-FU group compared to the control group (*p* < 0.001), with an approximate 1330-fold increase, indicating a strong inflammatory response induced by 5-FU. This expression was substantially reduced in the 5-FU + BBR (50 mg/kg) group (~ 107-fold increase; *p* < 0.01), reflecting the anti-inflammatory effect of low-dose BBR. In the 5-FU + BBR (100 mg/kg) group, TNF-α expression decreased further (~ 17-fold increase; *p* < 0.001), while the BBR-only group exhibited only a ~ 4-fold increase compared to the control, which was not statistically significant, suggesting minimal inflammatory activation under non-inflammatory conditions (Fig. 9). The data obtained from the fold changes were provided in supplementary Table 1.

#### *COX‑2* expression

*COX-2* expression followed a similar pattern, with an approximate 665-fold upregulation in the 5-FU group compared to the control group (*p* < 0.01), confirming the pro-inflammatory effect of 5-FU. In the 5-FU + BBR (50 mg/kg) group, *COX-2* expression was reduced to approximately 58-fold increase (*p* < 0.01), while the 5-FU + BBR (100 mg/kg) combination led to a further reduction to a 9-fold increase (*p* < 0.001), demonstrating a dose-dependent anti-inflammatory response. The BBR-only group exhibited a mild 5-fold increase, which was not significantly different from the control, indicating a limited effect under baseline conditions (Fig. 9).

#### Endothelial nitric oxide synthase (*eNOS*) expression

In contrast to the pro-inflammatory markers, *eNOS* expression was nearly undetectable in the 5-FU group compared to the control group (*p* < 0.001), indicating impaired endothelial and vasodilatory function. Treatment with 5-FU + BBR (50 mg/kg) resulted in a moderate upregulation of *eNOS* expression by approximately 5-fold (*p* < 0.01), while the 5-FU + BBR (100 mg/kg) combination significantly enhanced *eNOS* expression to approximately 68-fold (*p* < 0.001), indicating substantial vascular recovery. The BBR-only group showed a modest 4-fold increase, which was not statistically significant, suggesting limited endothelial stimulation under normal conditions (Fig. 9).

## Discussion

A variety of theories have been proposed to explain the pathophysiology of 5-FU-induced cardiotoxicity, including vasospasm, endothelial function impairment, and thrombogenic effects in coronary vessels, all of which contribute to cardiovascular complications^20^. Several studies have demonstrated that BBR possesses potential anti-arrhythmic and cardioprotective effects against doxorubicin-induced cardiotoxicity and isoproterenol-induced ischemic injury^14,21–23^.

5-FU intoxication led to a significant increase in the heart index. Conversely, co-treatment of intoxicated rats with BBR significantly mitigated the increase in relative heart weight caused by 5-FU administration. Histological examination of cardiac tissues revealed that this increase in relative heart weight was due to interstitial edema and inflammatory cell infiltration. In this context, Safarpour et al.^24^ reported that a single intraperitoneal dose of 5-FU at 100 mg/kg significantly reduced body weight in rats, attributing this effect to markedly reduced food intake, intestinal or liver injury, and a decrease in anaerobic gut bacterial colonies.

In the present study, the QRS duration was within the range reported in a previous study^25^. The 5-FU group exhibited significant alterations in ECG measurements, including prolonged QRS complex and PR interval durations, as well as elevated ST segments. These findings are consistent with a previous report describing similar changes associated with 5-FU-induced cardiotoxicity with a single dose of 125 mg/kg^26^. In addition, sinus arrhythmia and ventricular premature contractions (VPCs) were observed, corroborating earlier reports that 5-FU increases cardiac vulnerability to ventricular arrhythmias in rats^26^. Previous studies have demonstrated that BBR with 30 and 60 mg/kg for 10 days exerts cardioprotective effects against cardiac injury associated with diabetic heart and with 100 mg/kg for 10 days in isoprenaline-induced cardiac fibrosis in rats^27,28^. Similarly, concurrent administration of low or high doses of BBR with 5-FU alleviated, to varying extents, ECG abnormalities including prolonged QRS complex and PR interval durations, ST elevation, and arrhythmias. Notably, high-dose BBR exerted more pronounced cardioprotective effects, with heart rate, P amplitude, P duration, PR interval, and QRS amplitude returning to normal ranges.

Regarding hematological parameters, anemia is a common adverse effect of chemotherapeutic agents, and prolonged use of single-agent 5-FU can lead to first- and second-degree anemia in approximately 50% of cases^29^. Following a single IP injection of 5-FU (150 mg/kg), significant thrombocytopenia developed, followed by a stable and reversible thrombocytosis. Additionally, 5-FU is known to induce leukopenia, which can be mitigated with a glutamine-containing diet^30^.

Consistent with these findings and previous studies, our results showed significant reductions in RBC, WBC, and platelet counts, as well as lower Hb levels, in the 5-FU-treated group. However, the BBR-treated groups showed significantly improved reductions in RBC and platelet counts, as well as reduced Hb levels. Despite the known myeloprotective properties of BBR, neither treatment group was able to prevent the decline in WBC count. This may be attributed to the substantial myelosuppressive effect of 5-FU on granulocytic precursors, which may potentially outweigh the anti-inflammatory or antioxidant effects of BBR at the administered doses.

The significant increase in platelet count observed in both treatment groups relative to the control group may be attributed to the atheroprotective properties of BBR in conjunction with the effects of 5-FU. Yeager et al. reported that following a single IP dose of 5-FU (150 mg/kg), moderate thrombocytopenia was observed on day 7, followed by persistent rebound thrombocytosis between days 11 and 17. This rebound phenomenon necessitates close monitoring of platelet counts during the treatment period^65^.

Evaluation of cardiac biomarkers revealed that cTnI, a structural protein unique to the heart and considered organ-specific, though not disease-specific, serves as a reliable indicator of cardiomyocyte damage and toxicity^30^. CK-MB, a cytoplasmic and mitochondrial enzyme exclusive to myocardial tissue, is a marker of acute myocardial infarction. Additionally, the WHO has recommended CK-MB, AST, and LDH as part of the diagnostic panel for acute myocardial infarction (AMI)^31^.

Accordingly, myocardial damage induced by 5-FU was evidenced by a significant rise in cardiac biomarkers, including cTnI, CK-MB, and LDH. Concurrent administration of BBR mitigated this elevation, returning the levels of these biomarkers to near-normal values.

The reduction in LDH levels observed in the BBR-only group may be attributed to the protective effects of BBR against cellular injury, as LDH is present in nearly all body tissues. Moreover, special attention should be given to patients with LDH-A (glycogen storage disease) or LDH-B deficiency. Although glycogen storage disease is extremely rare in humans, further research should focus on dose optimization, particularly in the context of metabolic disorders.

Oxidative stress refers to an imbalance between the antioxidant and oxidant systems. MDA and antioxidant system indicators such as GSH, SOD, and TAC are routinely used to assess oxidative damage^32^. In the current investigation, 5-FU-induced oxidative damage in cardiac tissues was evidenced by a significant increase in lipid peroxidation products (MDA) and a decrease in the levels of GSH, SOD, and TAC. Conversely, BBR substantially reduced the 5-FU-induced redox imbalance in cardiac tissues. These findings suggest that the ameliorative effect of BBR against 5-FU-induced cardiotoxicity may be attributed to its ability to reduce oxidative stress.

Our results, align with previous studies demonstrate the antioxidant capacity of BBR in a dose of 50 mg/kg for seven days, which may be due to its ability to neutralize reactive oxygen species (ROS) and prevent cellular damage by enhancing the activity of endogenous antioxidant enzymes such as SOD, GSH, and TAC. BBR activates the AMP-activated protein kinase (AMPK) pathway, which regulates energy homeostasis and inhibits mitochondrial ROS production, thereby protecting cells from oxidative damage^33^. Additionally, BBR’s tetracyclic framework and quaternary ammonium group structures facilitate the direct scavenging of oxidative free radicals. Its rigid and aromatic structure further enhances its interaction with DNA and enzymes involved in oxidative stress regulation^33,34^.

Regarding the AST/ALT ratio in the 5-FU group compared to the control group, the ratio was nearly doubled in this study. Serum alanine transaminase (ALT) is primarily found in the liver, whereas serum aspartate transaminase (AST) is predominantly present in the liver, heart muscle, and other tissues. Liver cell damage is frequently assessed using the AST/ALT ratio^35,36^. Since the liver receives approximately 25% of the total cardiac output, it is susceptible to hemodynamic changes^37^. Consistent with other studies, we confirmed that the AST/ALT ratio increases in cardiac events, particularly those involving ST-segment elevation^38,39^. Co-treatment with BBR mitigated this effect by reducing AST levels by 6% in the low-dose treatment group and 5% in the high-dose treatment group (Table 3).

The elevation of AST levels in the BBR-only group relative to the control group may be attributed to significant hepatic metabolism or the modulatory effect of BBR on hepatic enzymes.

Furthermore, histological changes in heart tissue, including necrosis, vacuolation, and inflammatory cell infiltration, corroborated the biochemical findings. The 5-FU-treated group exhibited the highest degree of necrosis, vacuolation, and inflammatory cell infiltration. Similar findings, such as perivascular cellular infiltration and myocytic degeneration, have been reported in previous studies involving 5-FU administration of a single dose of 100 mg/kg^40^. However, concomitant administration of BBR at both doses significantly ameliorated these histopathological lesions.

Our findings are consistent with previous studies investigating the effects of BBR (60 mg/Kg) on doxorubicin- and isoproterenol-induced myocardial injury, where rats treated with BBR exhibited a reduced myocardial infarction area^22,23^.

Cardiac inflammatory and apoptotic markers, including TNF-α and caspase-3, were significantly increased in the 5-FU-treated group, consistent with earlier reports^41^. One of the key steps in apoptosis is the activation of caspases, particularly the cleavage of procaspase to active caspase. Caspase-3 functions as a central effector in many cell types, leading to DNase activation and subsequent DNA fragmentation^42^. 5-FU-induced cardiotoxicity enhances cardiac caspase-3 levels, promoting apoptosis and cell death. BBR significantly reduced caspase-3 levels and increased Bcl-2 expression in cardiac tissues. These findings align with previous reports confirming that BBR with a dose of 120 mg/kg for 14 days exerts anti-apoptotic effects by inhibiting caspase-3 activation and upregulating Bcl-2 expression^43^.

Moreover, the study demonstrated that BBR effectively increased the expression of the anti-apoptotic protein Bcl-2, while reducing the expression of the pro-apoptotic protein caspase-3^44^. Additionally, another study found that BBR attenuates myocardial ischemia-reperfusion injury by inhibiting the NF-κB signaling pathway^45^.

TNF-α is an inflammatory cytokine released by macrophages and monocytes. It is produced during acute inflammation and initiates a cascade of intracellular signaling events that ultimately lead to necrosis and apoptosis. TNF-α has been widely employed to investigate the inflammatory effects of chemotherapeutic agents such as cisplatin and doxorubicin. Cyclooxygenase-2 (COX-2) is an inducible enzyme that converts arachidonic acid into prostaglandins in response to inflammatory stimuli^46^. It has been demonstrated that BBR reduces the inflammatory response by inhibiting the production of pro-inflammatory cytokines, including IL-1β, IL-6, and TNF-α^33,47^.

In the present study, molecular analysis revealed that administration of 5-FU in rats significantly elevated the expression levels of *TNF-α* and *COX-2* genes. In contrast, BBR treatment significantly downregulated the expression of both markers in a dose-dependent manner. These findings indicate that BBR plays a crucial role in mitigating inflammation by suppressing the production of *TNF-α* and downregulating *COX-2* gene expression.

Endothelial nitric oxide synthase (eNOS) is a key enzyme responsible for nitric oxide (NO) production in vascular endothelial cells^48^. 5-FU and its metabolites exert direct toxic effects on myocytes and the vascular endothelium, including the inhibition of endothelial NO synthase. This downregulation of NO synthase is associated with coronary spasms and endothelium-independent vasoconstriction, mediated by the activation of protein kinases^49^.

Our findings confirmed that BBR upregulated *eNOS* gene expression levels, a result consistent with previous studies^50^. Furthermore, BBR was shown to alleviate endothelial dysfunction by activating the PI3K/Akt signaling pathway, which phosphorylates and activates *eNOS*, thereby enhancing NO production^51^.

The safety profile of BBR was previously evaluated by Hu et al.^52^, who reported that administration of 1500 mg/kg BBR for 12 weeks did not significantly alter complete blood count parameters, blood electrolytes, blood pressure, or ECG readings. These findings support the safety of BBR, even at high doses and over extended periods. Moreover, BBR has been shown to enhance the efficacy of several antineoplastic agents, including cisplatin, doxorubicin, and tamoxifen, across various types of cancer^53^.

In conclusion, this study investigated the role of BBR in mitigating the adverse effects of 5-FU-induced cardiotoxicity. The cardioprotective effects of BBR may be attributed to its antioxidant properties, which enable it to scavenge ROS, as well as its ability to regulate lipid peroxidation. At the molecular level, BBR may limit cardiac tissue injury by downregulating the gene expression of inflammatory cytokines such as COX-2 and TNF-α. At the cellular level, BBR’s cardioprotective properties can be attributed to its ability to modulate the mitochondrial apoptotic signaling pathway.

## Materials and methods

### Chemicals and reagents

Berberine chloride (primary reference standard, CAS No. 633-65-8) was purchased from Sigma-Aldrich Chemicals (St. Louis, MO, USA). 5-Fluorouracil (5-FU, Utoral 500 mg/10 mL) was sourced from Hikma Pharmaceutical Co. (6th of October, Egypt). Dimethyl sulfoxide (DMSO, HPLC grade, purity > 99.99%) was obtained from Sigma-Aldrich Co., St. Louis, MO, USA.

### Experimental animals

Fifty male Sprague-Dawley albino rats, approximately 8–9 weeks old and weighing 170 ± 30 g, were obtained from the Egyptian Company for Production of Vaccines, Sera, and Drugs (EGY VAC), Giza, Egypt. The animals were housed in the Animal House facility at the Department of Pharmacology, Faculty of Veterinary Medicine, Cairo University, under standard management conditions, with a 12-hour light/dark cycle, a temperature of 25 ± 2 °C, and a relative humidity of 45 ± 5%. Sawdust was used as bedding material, and the rats were provided free access to water and a nutritionally balanced chow diet (Nile Feed Company).

The selection of male animals in the study is commonly based on the potential confounding effects of the estrous cycle. The limited understanding of the potential influence of sex on the assessed outcomes likely contributes to the continuation of this practice^54^.

All experimental procedures were approved by the Ethics Committee of the Faculty of Veterinary Medicine, Cairo University, Egypt (Approval no. Vet CU110520251072), following the National Research Council’s Guide for the Care and Use of Laboratory Animals, the EU Directive 2010/63/EU for animal experiments, and the U.K. Animals (Scientific Procedures) Act, 1986, and related guidelines. Additionally, all techniques were documented in compliance with the ARRIVE guidelines^55^, ensuring the highest standards of animal welfare and scientific integrity.

### Experimental design

After two weeks of acclimatization under these conditions, to achieve a 95% power level and a 5% level of significance, the initial sample size was set at 10 rats per group. The sample size was determined using the Resource Equation method^56^. Rats were randomly assigned to five groups, each comprising 10 rats, based on their body weight range.

1. Group 1 (control negative group) received DMSO 2% only (as a vehicle) orally for 2 weeks.
2. Group 2 (cardiotoxic group) received a single IP injection of 5-fluorouracil (5-FU, 150 mg/kg) on the 1st day to induce cardiotoxicity. Refaie et al. demonstrated that a single IP dose of 150 mg/kg 5-FU corresponds to the human toxic dose^57^.
3. Group 3 (cardiotoxic and low-dose treatment group) received a single IP injection of 5-FU (150 mg/kg) on the 1st day, followed by oral administration of BBR (50 mg/kg) for 2 weeks (post-5-FU treatment)^34,58^.
4. Group 4 (cardiotoxic and high-dose treatment group) received a single IP injection of 5-FU (150 mg/kg) on the 1st day, followed by oral administration of BBR (100 mg/kg) for 2 weeks (post-5-FU treatment)^59,60^.
5. Group 5 (treatment-only group) received oral BBR (100 mg/kg) for 2 weeks.

Rats were closely monitored throughout the experiment for signs of discomfort, pain, distress, respiratory manifestations, changes in mucous membrane coloration, body weight fluctuations, morbidity, and mortality.

###  ECG evaluation

On the 14th day, all rat groups were anesthetized in an induction chamber containing 4% isoflurane (Isoflurane^®^; UP Pharma, Cairo, Egypt) and ambient air, followed by maintenance with 2% isoflurane in two liters of O_2_ via an anesthetic mask^61^. Rats were positioned in the supine posture, and surface ECG was recorded using limb electrodes. Lead II of the ECG was standardized for all measurements, including heart rate (HR), rhythm, duration, and amplitude of the P wave, duration and amplitude of the QRS complex, PR interval, and the position of the ST segment^25,26,62^.

### Estimation of relative heart weight

The rats in all groups were weighed on the first day before the injection and at the end of the treatment period (2 weeks after the first injection) to compare weight changes between the 1st and 14th day. Furthermore, the relative heart weight of the rats was determined using a digital scale as follows:

$$Relative \,heart\, weight=\frac{Rat\,heart\,weight}{Rat\,body\,weight}\times\,100\%$$

### Blood and tissue sampling

On the 15th day, blood samples were collected from the retro-orbital plexus. A portion of the blood was transferred into plain glass tubes and allowed to coagulate for 20 min at room temperature, followed by centrifugation at 3000 rpm for 10 min. The resulting serum was isolated and used for further biochemical analyses. Another portion of the blood was collected into EDTA tubes for subsequent hematological analysis.

Rats were euthanized by cervical dislocation, and heart tissues were excised and rinsed with ice-cold saline. Approximately 20–30 mg of the harvested heart tissue was immediately transferred to microtubes for the assessment of oxidative stress markers and part of the antioxidant enzyme activities. An additional 20–30 mg of heart tissue was placed into 1.5 mL RNase- and DNase-free microtubes and stored at − 80 °C for subsequent molecular analysis. The remaining tissue samples were placed in formalin-containing containers and, along with the serum samples, stored at − 20 °C for subsequent histopathological and immunohistochemical analyses.

### Hematological and biochemical assessment

#### Hematological parameters

Complete blood count (CBC), including hemoglobin (Hb, g/dL), red blood cell count (RBC, ×10^6^/µL), white blood cell count (WBC, ×10^3^/µL), and platelet count (PLT, ×10^3^/µL), was quantified using an automated hematology analyzer (Diatron).

#### Cardiac enzymes and markers

Cardiac troponin I (cTn1), creatine kinase-MB (CK-MB), and lactate dehydrogenase (LDH) enzyme activities were determined using standard methods with commercially available ELISA kits (SunLong Biotech, China), with catalog numbers SL0713Ra, SL0203Ra, and SL0434Ra, respectively.

#### Oxidative stress and antioxidant markers

Malondialdehyde (MDA), total antioxidant capacity (TAC), reduced glutathione (GSH), and superoxide dismutase (SOD) were determined using standard methods with commercially available kits from Biodiagnostic (Giza, Egypt).

#### Liver function enzymes

Alanine aminotransferase (ALT) and aspartate aminotransferase (AST) enzyme activities were determined using standard methods with commercially available kits from Chema Diagnostica.

### Histopathological and immunohistochemical assessment

#### Histopathological examination

Heart specimens were obtained and fixed in 10% neutral buffered formalin. Further processing was carried out using ascending concentrations of ethanol and xylene. Tissues were embedded in paraffin, sectioned with a rotary microtome into four µm-thick sections, and stained with hematoxylin and eosin. Specimens were assessed and examined in 10 randomly selected fields per section at high magnification. A light microscope equipped with a digital camera was used for tissue examination and photography. The pathologist was blinded to the treatments of different groups.

#### Immunohistochemical examination

Paraffin-embedded tissue sections were deparaffinized, rehydrated, and placed in citrate buffer (pH = 6) in a microwave for 16 min. After washing with TBS, primary antibodies against caspase-3 (Abexxa, UK) and Bcl-2 (Abexxa, UK) were applied to the slides and incubated overnight. A secondary biotinylated antibody and the avidin-peroxidase complex (universal kit, Bio-SB, USA) were applied, followed by DAB substrate for color detection, according to the manufacturer’s protocol. The percentage of positive brown staining in the tissue was quantified using ImageJ software in five images per animal at 200× magnification.

#### Statistical analysis

The area percentage of positively stained brown tissue was analyzed using SPSS software to detect statistical significance between groups through ANOVA, followed by post-hoc tests (Duncan’s test and Tamhane’s test).

### RNA extraction and real-time polymerase chain reaction (RT-PCR)

#### RNA extraction and quantification

Total RNA was extracted from cardiac tissue using the RNeasy Mini Kit (Qiagen, Germany, #74904) according to the manufacturer’s protocol. RNA purity and concentration were assessed using a NanoDrop spectrophotometer (NanoDrop Technologies, Wilmington, DE), with an acceptable A260/A280 ratio ranging from 1.8 to 2.1. RNA integrity was evaluated by electrophoresis on a 1.5% agarose gel.

#### cDNA synthesis

One microgram of total RNA was treated with RNase-free DNase I (Thermo Scientific™, USA, #EN0521) to eliminate genomic DNA contamination. First-strand complementary DNA (cDNA) was synthesized using the RevertAid First Strand cDNA Synthesis Kit (Thermo Scientific™, USA, #K1622) with random hexamer primers. The reaction mixture contained 250 ng of RNA, 4 µL of 2× enzyme buffer, 1 µL of RiboLock RNase Inhibitor, 1 µL of 10 mM dNTP mix, and 2 µL of RevertAid M-MuLV RT enzyme, with the final volume adjusted to 20 µL using DEPC-treated water. To ensure the complete removal of genomic DNA, a no-reverse transcriptase (no-RT) control was included by omitting the reverse transcriptase enzyme from the cDNA synthesis reaction. This control was processed in parallel with the test samples to monitor potential genomic DNA contamination.

The mixture was incubated in a PCR thermocycler under the following conditions: 5 min at room temperature for primer annealing, 42 °C for 60 min for reverse transcription, and 70 °C for 5 min to terminate the reaction. The synthesized cDNA was stored at -80 °C for further analysis.

#### Quantitative real-time PCR (RT-qPCR)

Quantitative real-time PCR was conducted using the ABI StepOnePlus 7500 Real-Time PCR System (Applied Biosystems, USA) with Maxima SYBR Green qPCR Master Mix (Thermo Scientific, USA, #K0251). Each reaction mixture (25 µL final volume) contained 12.5 µL of 2× SYBR Green Master Mix, 0.5 µM of each primer, 1 µL of cDNA, and nuclease-free water.

The thermal cycling conditions were as follows: 95 °C for 10 min (initial denaturation), followed by 40 cycles of 95 °C for 15 s, 60 °C for 30 s, and 72 °C for 1 min (amplification).

Melt curve analysis was performed to confirm reaction specificity and the absence of primer-dimer formation. Gene expression levels of *TNF-α*, *COX-2*, *eNOS*, and the reference gene *GAPDH* were quantified using the comparative cycle threshold (ΔCt) method. Relative fold changes were calculated using the following formula:

$$Relative\,fold\,change={2}^{-\left({\Delta}Ct\right)}$$

Cycle threshold (Ct) values were determined by automated threshold analysis. All reactions were conducted in technical triplicate, and five independent biological replicates were analyzed. Primer sequences were designed based on previously published sequences. Primer sequences and amplicon sizes are provided in Table 3.

#### Statistical analysis

Relative mRNA expression levels were compared to the reference gene *GAPDH* using the ΔCt method. Data were presented as mean ± standard error (SE). Statistical significance was evaluated using an unpaired independent t-test to compare differences between groups. A two-tailed t-test was performed to assess significance, and a p-value of < 0.05 was considered statistically significant. All statistical analyses were conducted using GraphPad Prism (8.0.2, GraphPad Software, CA, USA) (Table 3).

**Table 3:** Primer sequences and amplicon lengths are used for gene expression.

Gene name	Sequence		Amplicon Length^e^ (bp)	Reference
GAPDH	F/W	GTATTGGGCGCCTGGTCACC	202	^63^
	R/W	CGCTCCTGGAAGATGGTGATGG		
TNF-α	F/W	AAATGGGCTCCCTCTCATCAGTTC	111	^63^
	R/W	TCTGCTTGGTGGTTTGCTACGAC		
COX-2	F/W	TGTATGCTACCATCTGGCTTCGG	83.8	^63^
	R/W	GTTTGGAACAGTCGCTCGTCATC		
eNOS	F/W	CGAGATATCTTCAGTCCCAAGC	328	^64^
	R/W	GTGGATTTGCTGCTCTCTAGG		

## Electronic supplementary material

Below is the link to the electronic supplementary material.

Supplementary Material 1

## Data Availability

The datasets used and/or analyzed during the present study are available from the corresponding author upon reasonable request.
