# Supplementary material for: Ameliorative effects of Berberine chloride against 5-fluorouracil-induced cardiotoxicity in Sprague Dawley rats
Source: Sci Rep. 2025 Aug 2;15:28276. doi: 10.1038/s41598-025-12389-6 (PMC12318004; doi:10.1038/s41598-025-12389-6)
Supplement: Supplementary file 1 — Supplementary Material 1 [file 41598_2025_12389_MOESM1_ESM.docx]

| Relative fold changes between groups | 5-FU vs control group | 5-FU + BBR (50 mg/kg) vs control group | 5-FU + BBR (100 mg/kg) vs control group | BBR (100 mg/kg) only vs control group |
| --- | --- | --- | --- | --- |
| TNF-α gene expression | ~1330-fold | ~107-fold | ~17-fold | ~4-fold |
| COX-2 gene expression | ~665-fold | ~58-fold | ~9-fold | ~5-fold |
| eNOS gene expression | ~0-fold | ~5-fold | ~68-fold | ~4-fold |

**Supplementary table. 1** The effect of BBR administration on *TNF-α*, *COX-2*, and *eNOS* gene expression levels with relative fold changes among the groups.
